# Green “turn-off” luminescent nanosensors for the sensitive determination of desperately fluorescent antibacterial antiviral agent and its metabolite in various matrices

**DOI:** 10.1038/s41598-023-40946-4

**Published:** 2023-08-29

**Authors:** Hadil M. Elbardisy, Mai M. Elnaggar, Tarek S. Belal, Mahmoud A. Ragab, Amira F. El-Yazbi

**Affiliations:** 1 Pharmaceutical Analysis Department, Faculty of Pharmacy, Damanhour University, Damanhour, 22511 Egypt; 2 Department of Pharmaceutical Analytical Chemistry, Faculty of Pharmacy, Alexandria University, Alexandria, 21521 Egypt; 3 Department of Pharmaceutical Chemistry, Faculty of Pharmacy, Damanhour University, Damanhour, Buhaira 22516 Egypt

**Keywords:** Analytical chemistry, High-throughput screening, Drug discovery and development, Drug development, Fluorescence imaging, Nanoparticles

## Abstract

Nitazoxanide (NTX) is an antimicrobial drug that was used for the treatment of various protozoa. However, during the coronavirus pandemic, NTX has been redirected for the treatment of such virus that primarily infect the respiratory tract system. NTX is now used as a broad-spectrum antiviral agent. In this study, a highly sensitive and green spectrofluorometric method was developed to detect NTX in various dosage forms and its metabolite, tizoxanide (TX), in human plasma samples using nitrogen and sulfur co-doped carbon quantum dots nanosensors (C-dots). A simple and eco-friendly hydrothermal method was used to synthetize water soluble C-dots from citric acid and l-cysteine. After excitation at 345 nm, the luminescence intensity was measured at 416 nm. Quenching of C-dots luminescence occurred upon the addition of NTX and was proportional to NTX concentration. Assessment of the quenching mechanism was performed to prove that inner filter effect is the underlying molecular mechanism of NTX quenching accomplished. After optimizing all experimental parameters, the analytical procedure was evaluated and validated using the ICH guidelines. The method linearity, detection and quantification limits of NTX were 15 × 10^–3^–15.00 µg/mL, 56.00 × 10^–4^ and 15 × 10^–3^ µg/mL, respectively. The proposed method was applied for the determination of NTX in its commercial pharmaceutical products; Nanazoxid^®^ oral suspension and tablets. The obtained % recovery, relative standard deviation and % relative error were satisfactory. Comparison with other reported spectrofluorimetric methods revealed the superior sensitivity of the proposed method. Such high sensitivity permitted the selective determination of TX, the main metabolite of NTX, in human plasma samples making this study the first spectrofluorimetric method in literature that determine TX in human plasma samples. Moreover, the method greenness was assessed using both Eco-Scale and AGREE approaches to prove the superiority of the proposed method greenness over other previously published spectrofluorimetric methods for the analysis of NTX and its metabolite, TX, in various dosage forms and in human plasma samples.

## Introduction

**Figure 1:**
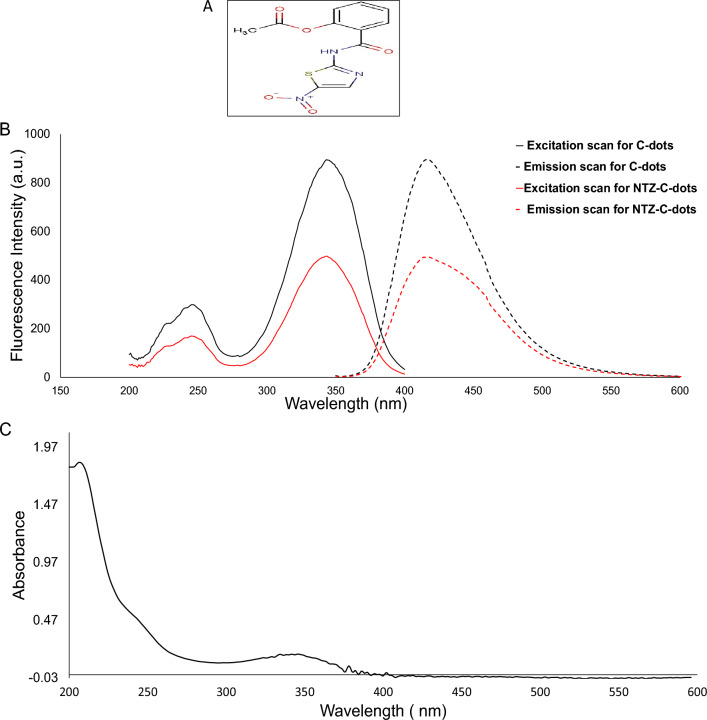
(**A**) Chemical structures of Nitazoxanide (NTX), (**B**) Excitation (solid line) and emission spectra (dashed line) of C-dots in the presence (red line) and in absence (black line) of 5 µg/mL of NTX (λ emission = 416 nm and λ excitation = 345 nm) and (**C**) UV–Visible absorption spectrum of C-dots.

Nitazoxanide (NTX), (2-[(5-nitro-1,3-thiazol-2-yl) carbamoyl]phenyl] acetate) (Fig. 1a) is a synthetic oral broad-spectrum antiparasitic medication that was first synthesized in the 1980s. NTX was proven to be effective against human helminths, intestinal protozoa, anaerobic bacteria and viruses. It is used in the management of infections caused by *Cryptosporidium parvum, Giardia lamblia* or *Clostridium difficile*^1–4^. It is also given as a single drug in the management of Helicobacter pylori bacteria^5^. Both RNA and DNA viruses are highly susceptible to its activity, such as hepatitis B and C, influenza A and Coronaviruses (MERS, SARS, SARS-CoV-2)^6,7^. Additionally, since the declaration of coronavirus as a worldwide pandemic by the WHO on March 12, 2020^8^, different studies were made to investigate its effectiveness towards the treatment of COVID 19. It has been demonstrated that remdesivir and NTX work together to treat acute SARS-CoV-2 infection, enhancing viral clearance and lowering the risk of developing resistance^9^. On the other hand, NTX/azithromycin combination was proven to treat the life-threatening cytokine storm^10^. Also, the use of NTX, ribavirin and ivermectin in addition to zinc capsules for treatment of COVID19 effectively removed the SARS-COV2 from the nasopharynx faster than symptomatic therapy^11^. NTX seems to be effective against SARS-CoV-2 in pregnancy without causing undesirable side effects for the fetus^7^. With respect to the original SARS-CoV-2 Wuhan-spike and several newly developing variants, such as the Delta variant, NTX was able to significantly reduce the viral burden^12^, so it was proven to be safe and affordable treatment for COVID 19.

Recent review of literature showed that many analytical techniques have been employed for the quantification of NTX, such as, spectrophotometry^13–15^, high performance liquid chromatography (HPLC)^16,17^, liquid chromatography- mass spectrometry (LC–MS)^18^, and electrochemically using voltammetric technique^19^. Optically, NTX has an absorption peak at 345 nm, however, it does not possess any native fluorescence. To the best of our knowledge, two spectrofluorimetric studies have been reported for the quantification of NTX. Abdel-Lateef et al.^20^ has determined NTX in tablets or its metabolite in human urine samples using sodium hypochlorite which oxidizes NTX to a highly fluorescent product. While, Qandeel et al.^21^ have used plant synthesized quantum dots for the analysis of NTX in capsules dosage forms.

Fluorescent carbon quantum dots are unique nanosensors with size-dependent optical and electrical features^22^. Carbon dots have distinct benefits due to their unique optical properties, extremely minute size and high surface-to-volume ratio, such as tunable strong fluorescence starting from deep ultraviolet to near infrared, stability against photo bleaching and photo blinking, additionally they are able to absorb light at a broad bandwidth and emit it at a narrow spectrum. Carbon dots are chemically inert, eco-friendly, and resistant to metabolic decomposition in bio-applications, it is also small hazardous compared with conventional semiconductor-based dots^23,24^. Many reported methods have been used for the synthetization of fluorescent carbon quantum dots, including: chemical oxidation^25,26^, hydrothermal cutting strategies^27^, electrochemical oxidation^28,29^, carbonizing from organics routes^30,31^ and microwave-assisted procedure^21^. However, most of the aforementioned methods require complicated equipment, difficult procedures and didn’t produce a high yield, so they are not preferred.

To date, numerous publications found in the literature have employed carbon dots synthesized by creative methodologies in many pharmaceutical applications, these include: the use of green one-pot synthesized nitrogen and sulfur co-doped carbon quantum dots for the determination of salinomycin and maduramicin in food samples^32^. Microwave-assisted prepared nitrogen-doped carbon quantum dots utilized for cellular imaging and detection of palbociclib in living cancer cells^33^. One-pot hydrothermal nitrogen and sulfur-doped carbon quantum dots for the determination of olanzapine and diazepam in biological fluids, dosage forms and application to content uniformity testing^34^. A Green microwave-assisted synthesized nitrogen-doped carbon quantum dots, using orange juice as a carbon source and urea as a nitrogen source for the quantification of the anticancer drug dacomitinib in bulk and in a pharmaceutical dosage form^35^. Citric acid and thiosemicarbazide were used as precursors for hydrothermal preparation of sulfur and nitrogen doped carbon quantum dots for spectrofluorimetric estimation of gliclazide and saxagliptin^36^, some nitro containing compounds^37^, and other applications^38^.

In this study, nitrogen and sulfur co-doped quantum dots (C-dots) were synthesized from citric acid and l-cysteine via a simple one-step hydrothermal method where citric acid serves as the carbon source, while l-cysteine is the source of nitrogen and sulfur^24^. The synthesized C-dots were successfully used for the sensitive determination of NTX in various dosage forms and tizoxanide (TX), the main metabolite of NTX, in human plasma samples. NTX is immediately and completely metabolized by deacetylation to an active metabolite TX after its oral administration in human^17,39^. Also, TX is the only metabolite detected in feces (two-third of NTX dose). In addition, TX was found to be the only species obtained by incubation with human microsomes^39^. Following administration of a 500 mg NTX oral dose, TX has a C_max_ of 1.9 mg/L; two to six hours after dosing and a terminal half-life ranging from 1.03 to 1.6 h^40^. Up to our knowledge, no spectrofluorimetric method was reported previously to determine TX in human plasma samples. The proposed method was simple and highly reproducible for the quantification of NTX and TX with high selectivity and sensitivity. Such high sensitivity permitted the simple determination of TX in human plasma samples. Furthermore, the development of green analytical methods has recently been one of the main focus of research^41–46^. For this purpose, the use of the analytical eco-scale protocol^47^ and analytical GREEnness (AGREE) approach^48^ were accomplished to clarify the greenness of the proposed method, specifically, in regard to the consumption of energy, the creation of waste and dangerous chemicals. After comparison of the proposed method to other reported spectrofluorimetric methods for the analysis of NTX, our proposed method proved to have better sensitivity for the determination of NTX, having the benefit of being simpler, less expensive and greener. Therefore, our proposed method can be readily used for quality control and bioavailability purposes as a simple, eco-friendly and efficient analytical tool.

## Experimental

### Materials and reagent

Nitazoxanide (NTX) was supplied by Alandalous medical company, Egypt. Its purity was certified to be (99.7 ± 0.72%). Anhydrous citric acid (99.9%) and l-cysteine (98%) were purchased from Loba—Chemie (Mumbai, India). Analytical reagent grade of boric acid, disodium hydrogen phosphate, sodium hydroxide, phosphoric acid, sulphuric acid, hydrochloric acid, methanol, ethanol, acetone, isopropyl alcohol and acetonitrile were obtained from El-Nasr chemical company, Cairo, Egypt. Surface active agents, cetrimide, Tween 80 and sodium dodecyl sulfate were supplied from Sigma Aldrich, Germany. Plasma samples were kindly supplied from El-Shatby University Hospital, Blood Bank, Alexandria, Egypt, and were stored frozen until use. Fresh deionized water was used throughout the work. Pharmaceutical preparations involved in this study are Nanazoxid^®^ oral suspension labeled to contain 100 mg NTX/5 mL and Nanazoxid^®^ film coated tablets labelled to contain 500 mg NTX (Utopia Company for Pharmaceuticals Industry, Cairo, Egypt).

### Instrumentation and characterization

Spectrofluorometric measurements were carried out using a Cary Eclipse fluorescence spectrophotometer (Agilent technologies, USA (model: G9800A)). The instrument was equipped with 1 cm quartz cell and 150 W xenon lamp. A Shimadzu UV–Vis light spectrophotometer (USA model 1800) was utilized and Fourier transform infrared (FT-IR) spectroscopy spectra were measured utilizing Cary 360 FT-IR (Agilent technologies, USA). TEM-1400 plus electron microscope was used to examine the synthesized C-dots morphology. A thermo Heratherm OGS60 oven and hydrothermal autoclave reactor 100 mL stainless steel 316 grade A PTFE were also used in synthetization of carbon dots reagent. Additionally, centrifuge PLC series. Model: PLC-03, power: 220 V/50 Hz; 0.65 A. Gemmy Industrial Crop. was used. All pH measurements were recorded using Crison instruments SA (Barcelona, Spain) and sonication was made using Soltec soluzioni technology sonicator (Italy, model:2200EP). All fluorimetric measurements were performed at room temperature.

### Synthesis of C-dots

Anhydrous citric acid (1.82 g, 9.5 mmol/L) and l-cysteine (1 g, 8.3 mmol/L) had been dissolved in 10 mL deionized water, and the mixture was then evaporated at 70 °C for 12 h to generate a thick syrup. This thick syrup was then transferred into a 100 mL Teflon-lined stainless-steel hydrothermal autoclave reactor. Afterwards, the mixture was heated hydrothermally in a heating oven at 200 °C for 3 h at a rate of 10 °C min^−1^. The reaction mixture was then allowed to cool at room temperature for several hours and the black syrup product was neutralized with 1M NaOH solution and diluted with deionized water to 100 mL. Finally, it was ultra-sonicated for 5 min at room temperature before filtration^24^.

### Preparation of stock solution

NTX stock solution (500 μg/mL) was prepared in acetonitrile. Deionized water was used as a green solvent to dilute the drug solution to prepare another diluted stock solution of 5 μg/mL, by transferring 0.5 mL in 50 mL volumetric flask. Deionized water is also used to obtain the final working solutions by transferring 0.1 mL from C-dots reagent and different volumes from the drug stock solution were added into a set of 10-mL volumetric flasks and completed to volume with deionized water.

### Construction of the calibration curve

To construct a calibration curve for NTX, working solutions were prepared by transferring accurate volumes from the NTX standard stock solution covering the concentration range 15 × 10^–3^–15.00 µg/mL into a set of 10-mL volumetric flasks and 0.1 mL of C-dot solution was added to each flask. The solutions were mixed and the volume was completed to the mark using deionized water. The fluorescence intensity of the working solutions was measured at λ emission = 416 nm (λ excitation = 345 nm). The readings were deduced from the corresponding reading of a blank that had received the same treatment. A calibration graph was constructed by plotting the difference in fluorescence intensity against the corresponding NTX concentrations (μg/mL).

### Application to commercial pharmaceutical dosage forms

#### Analysis of Nanazoxid^®^ oral suspension

To create a solution with a final concentration of 500 μg/mL, a volume of 2.5 mL of Nanazoxid^®^ oral suspension were accurately transferred to a 100 mL volumetric flask. To extract NTX, 50 mL of acetonitrile were added to the oral suspension, sonicated for 15 min, left to cool, completed to the mark with the same solvent (acetonitrile) and then filtered. Afterwards, precise quantities from the filtered solution were diluted with deionized water to obtain sample solutions with concentrations within the linearity range.

#### Analysis of Nanazoxid^®^ film coated tablets

Twenty Nanazoxid® tablets labeled to contain 500 mg NTX were weighed to calculate the average weight of tablets. After the tablets were ground, the average weight of the powder was transferred to a 100 mL volumetric flask. To extract the drug, 50 mL of acetonitrile were added to the powder, sonicated for 15 min, left to cool, completed to the mark with acetonitrile and then filtered. In a 100-mL volumetric flask, 10 mL of the filtered solution were transferred and diluted with acetonitrile to obtain a stock solution of the drug with final concentration of 500 µg/mL. Deionized water was used to dilute the previous solution to obtain sample solutions of final concentrations within the linearity range indicated above, and they were analyzed using the procedure described under “Construction of the calibration curve”. Finally, the percentage recoveries were computed.

### Tizoxanide preparation

The method of Shalan et al.^17^ was adapted for TX preparation. TX was obtained by acid hydrolysis of NTX where 50 mL of 1 M HCl were added to 50 mg of NTX. The mixture was refluxed for 3 h at 100 °C. TX preparation was confirmed by spotting on silica gel 60F_254_ TLC plates and developed with chloroform: methanol: NH_3_ solution: acetic acid in the ratio 95:5:1:1 v/v and pH 5.8. The precipitate formed was filtered and allowed to dry at room temperature. The dried powder was then used in the following experiments.

### Analysis of NTX and its metabolite (TX) in spiked human plasma

Standard calibration curves were plotted for human plasma spiked with varying amounts of NTX or TX. 200 μL aliquots of plasma were transferred into a series of centrifugation tubes. Aliquots of standard solution of NTX or TX were added to give a final concentration within the range 0.015–5 μg/mL. The mixtures were vortexed and 0.5 mL acetonitrile was added for protein precipitation. The tubes were vortexed for 1 min and then centrifuged at 3000 rpm for 3 min. The clear supernatant was then quantitatively transferred into 5-mL volumetric flasks and completed to volume with deionized water. Calibration curves were constructed using the procedure described under “Construction of the calibration curve”. A blank experiment was simultaneously prepared. The difference in fluorescence intensity was then plotted against the final concentration to obtain the calibration curve. Consequently, the corresponding regression equations were derived. Samples of human plasma spiked with different amounts of NTX or TX were prepared as described above and the recovered contents of each drug was quantified using its corresponding regression equation.

## Results and discussion

### Spectral characteristics and optimization of the proposed methods

#### Optical characteristics of the synthesized C-dots

The fluorescence quantum yield of the synthesized C-dots was accomplished by using quinine sulphate as a reference, activated with 345 nm UV light. It is computed to be 73.0%^24^. While upon using 4′,6-diamidino-2-phenylindole (DAPI) dissolved in dimethyl sulfoxide as a second standard, the quantum yield is calculated to be 71.2%. These findings demonstrate that the prepared C-dots exhibit relatively high quantum yield (at least 70%)^24^.

The optical characteristics of the synthesized C-dots were evaluated using fluorescence spectroscopy and UV–Vis absorption. The fluorescence of the synthesized C-dots was measured. Figure 1b demonstrates its fluorescent spectrum at an emission wavelength of 416 nm after excitation at 345 nm corroborating with Abd Elhaleem et al*.* published work^22^. While upon addition of 5 µg/mL of NTX to the C-dots solution, significant quenching of the C-dots fluorescence occurs as illustrated in Fig. 1b. Moreover, the UV–Visible absorption spectrum of the prepared C-dots was recorded (Fig. 1c) showing two main peaks at 212 and 345 nm.

**Figure 2:**
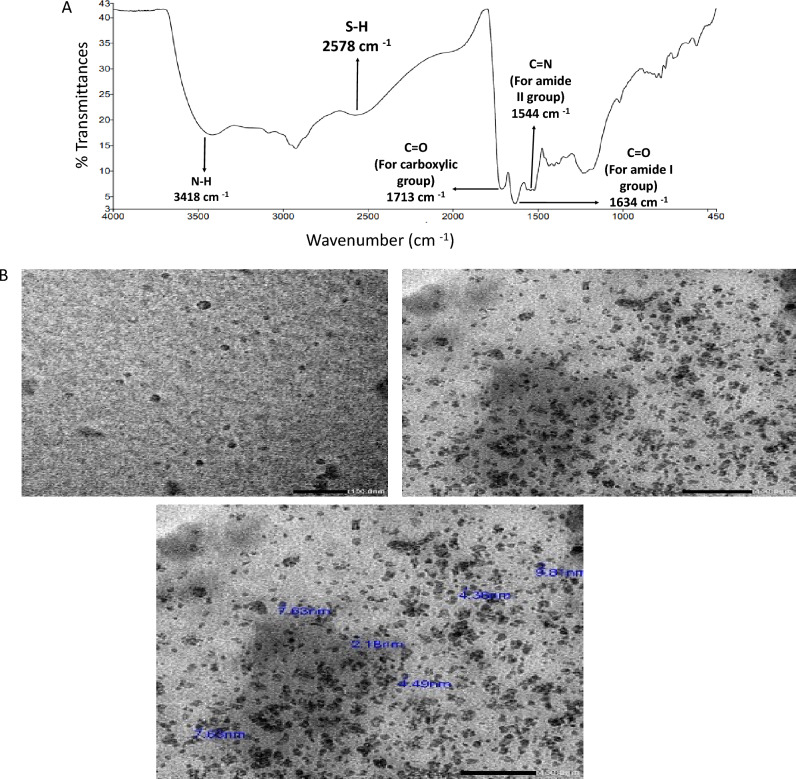
(**A**) FTIR spectrum of the prepared C-dots and (**B**) TEM images of C-dots.

In order to confirm the structure of the synthesized C-dots, Fourier transform infrared (FTIR) spectrum of the C-dots was measured. Results are demonstrated in Fig. 2a. The Fourier transform infrared (FTIR) spectrum of the synthesized C-dots showed N–H stretching vibration at 3418 cm^−1^. The Carboxylic O–H stretching vibration was accounted for by a very broad absorption band from 3400 to 2500 cm^−1^. The S–H stretching vibration was observed at 2578 cm^−1^. The C=O stretching vibration for carboxylic acid was observed at 1713 cm^-1^ and for amide carbonyl (Amide I) at 1634 cm^−1^. The C=N stretching vibration (Amide II band) was assigned to 1544 cm^-1^. In addition, TEM imaging was performed to explore the morphological features and size of the C-dots. Figure 2b shows that the synthesized C-dots are sphere-shaped and the range of the particle size distribution was 2.18–9.8 nm.

#### Elucidation of the quenching mechanism

**Figure 3:**
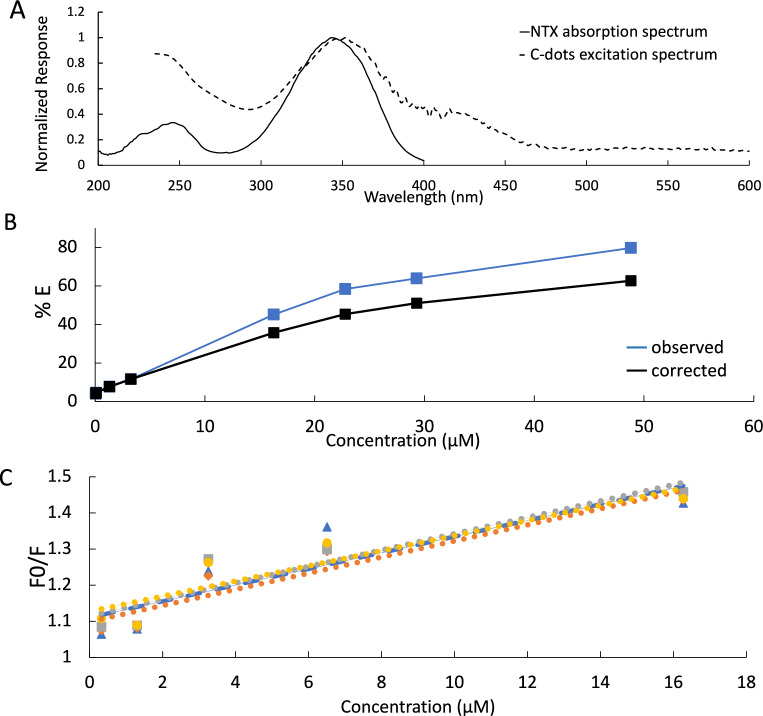
(**A**) Absorption spectrum of NTX overlayed on the excitation spectram of C-dots, (**B**) Efficiency (%E) of observed and corrected fluorescence of C-dots after addition of different concentrations of NTX and (**C**) Stern–Volmer plot of the interaction of NTX with C-dots at different temperatures (25 °C, 30 °C, 35 °C, 40 °C).

There are several methods that can result in fluorescence quenching, such as dynamic quenching, static quenching, and the inner filter effect (IFE)^37^. In this study, the absorption spectrum of the drug showed high extent of overlapping with the excitation spectrum of the prepared C-dots. This is demonstrated in Fig. 3a. Therefore, IFE is possible to be the quenching mechanism^37^. In order to investigate the effect of inner filter effect, the corrected fluorescence intensity was calculated according to the following equation^35,37^:

$${\text{F}}_{{{\text{corrected}}}} = {\text{ F}}_{{{\text{observed}}}} \times { 1}0^{{\left( {{\text{Aex}} + {\text{Aem}}} \right)/{2}}}$$

where F_corrected_ is the corrected fluorescence intensity after removal of IFE, F_observed_ is the observed fluorescence intensity, and Aex and Aem are the absorbance of NTX at the excitation and emission wavelengths of the C-dots, respectively.

Following this, the suppressed efficiency (%E) for the corrected and observed fluorescence intensity was calculated using the following equation^35,37^.

$$\% {\text{E }} = \, \left[ {{ 1 } - \, \left( {{\text{F}}/{\text{ F}}_{0} } \right)} \right] \, \times { 1}00$$

where F and F_0_ are the emission intensities of the NTX-C-dots mixture and C-dots alone, respectively. A plot of %E versus NTX concentration was constructed. Results illustrated in Fig. 3b demonstrated loss in suppression efficiency that confirms that IFE is the main trigger of quenching of C-dots fluorescence by NTX.

Other mechanisms besides IFE may take place, Stern–Volmer equation was used to determine the other possible mechanisms that could be responsible for the quenching of C-dots native fluorescence^35,37^.

$${\text{F}}_{0} /{\text{ F }} = { 1 } + {\text{ K}}_{{{\text{sv}}}} \left[ {{\text{NTX}}} \right]$$

where F_0_ and F represent the fluorescence intensities in the absence and presence of drug, respectively, K_sv_ represents the Stern–Volmer quenching constant and [NTX] is the drug molar concentration.

Both static and dynamic quenching happen due to molecular interaction between the fluorophore and the quencher. In dynamic quenching, during the excited state, the quencher must diffuse to the fluorophore. The molecules don't alter permanently as a result of the quenching. While in static quenching, a non-fluorescent compound between the quencher and the fluorophore is created. The quenching may result through excited-state processes, ground-state complex creation, molecular rearrangements, collisional quenching, energy/electron transfer, and emission group destruction^49,50^.

**Figure 4:**
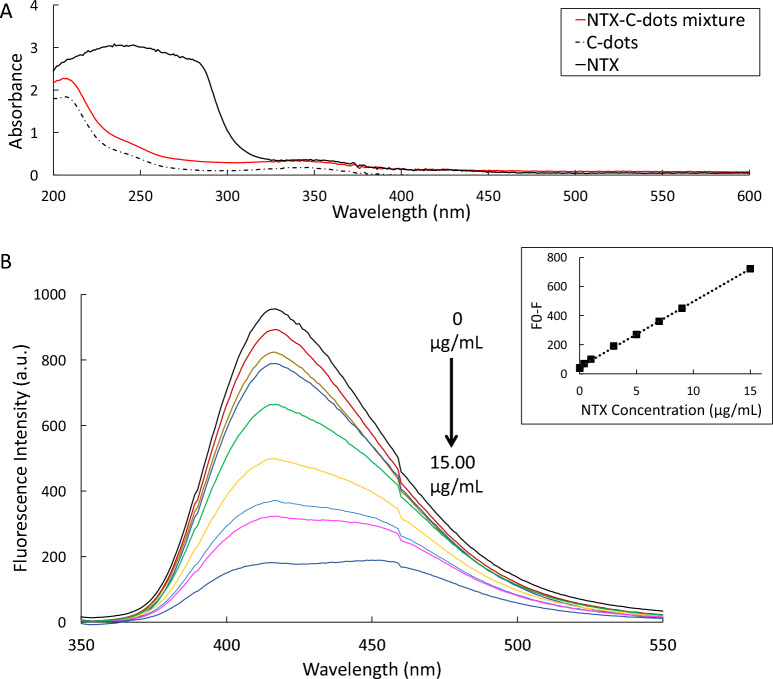
(**A**) Absorption spectra of C-dots, NTX and NTX/C-dots mixture and (**B**) Fluorescence emission spectra of 0.1 mL C-dots in aqueous solution upon addition of various NTX concentrations (0.0–15.0 μg/mL). The inset represents the corresponding calibration curve.

In order to distinguish between static quenching and dynamic quenching, the temperature dependency of the Stern–Volmer plot was explored^50^. K_sv_ value increases with increasing the temperature in case of dynamic quenching while, it decreases with increasing the temperature in case of static quenching^37^. Collisional quenching takes place at higher temperatures as diffusion occurs more rapidly. On the other hand, static quenching is generally less effective at higher temperatures due to the dissociation of weakly bound complexes^50^. For this purpose, five different NTX concentrations were measured at several temperatures (25 °C, 30 °C, 35 °C, 40 °C) and the obtained Stern–Volmer plots were compared (Fig. 3c). Results, demonstrated in Fig. 3c, show that the K_sv_ constant obtained from all plots were ~ 0.022 L mol^−1^ and were not affected by the temperature increase. As a result, both static and dynamic quenching are excluded, leaving IFE as an expected quenching mechanism^21^. For further confirmation of the elucidated mechanism UV absorption spectra for NTZ, C-dots and the NTZ-C-dots mixture were plotted (Fig. 4a) and no new absorption peaks were observed which emphasizes that the quenching mechanism is not a static quenching^33,35^.

### Optimization of reaction conditions

The reaction conditions were investigated to obtain the highest quenching of the analyzed drug. Various experimental factors were optimized. The studied parameters were: type and pH of the buffer, buffer volume, effect of adding different surfactants, reaction time and the effect of the diluting solvent.

#### Buffer type and pH

Both borate (25 mM) and phosphate (25mM) buffers were examined. The effects of different pHs for both buffers (pH 3, 5, 7 and 10) (Figs. S1a and b in supplementary information) and different volumes of borate buffer pH 7 (2–8 mL in 2 mL increments) (Fig. S1c in supplementary information) on NTX-C-dots quenching were investigated and were compared to the quenching of aqueous solutions NTX-C-dots. Results demonstrated in Fig. S1 show no considerable enhancement in C-dots quenching using either buffers. Therefore, the pH had no significant impact on the C-dots quenching by the studied drug. Accordingly, deionized water will be used as the diluting solvent. This will increase the simplicity and greenness of the proposed method.

#### Effect of surfactants

Previously published study reported the synergistic effect of sodium dodecyl sulfate surfactant on the fluorescence of C-dots^51^. So different cationic, anionic and nonionic surfactants were added to the measured solutions to examine the quenching effect of NTX. Cetrimide, sodium lauryl sulfate (SLS) and Tween 80 were selected as cationic, anionic and nonionic surfactants, respectively. Different concentrations were tried to study the surfactant effect, by changing concentration from 0.002 to 0.008 M for cetrimide (Fig. S2a in supplementary information), from 0.005 to 0.02 M for SLS (Fig. S2b in supplementary information) and from 0.2% to 0.8% for Tween 80 (Fig. S2c in supplementary information). The fluorescence intensity of NTX-C-dots was recorded at each concentration and was compared to the quenching of aqueous solutions NTX-C-dots. Results demonstrated in Fig. S2 show that the best quenching effect of NTX was achieved without adding any surfactants. Therefore, no surfactant was added to the proposed method which enhance the simplicity and greenness of the method.

#### Effect of diluting solvent

The effect of diluting solvents was investigated for the fluorimetric analysis. Different solvents such as: methanol, ethanol, isopropyl alcohol, acetonitrile, acetone, 0.1 M HCL, 0.1 M H_2_SO_4_, 0.1 M H_3_PO_4_, 0.1 M NaOH and deionized water were tested. As illustrated in Fig. S3a in supplementary information, deionized water gave the best quenching effect for the C-dots and maximizes the assay sensitivity. This may be attributed to the fact that more polar solvents, such as deionized water, reduce the π–π* transitions energy and maximize the n–π* transition energy, resulting in enhancement of the reagent fluorescence intensity^52^. Thus, deionized water was chosen as the diluting solvent for the assay and this made the method simpler, with lower cost and more importantly, ecofriendly.

#### Effect of reaction time

The effect of the reaction time upon the addition of NTX to C-dots was tested at various time intervals from zero to 30 min. The fluorescence intensity of NTX-C-dots mixture was measured every 5 min (Fig. S3b in supplementary information). It was observed that instantaneous reaction takes place immediately (at zero time) upon addition of the drug to the C-dots. Furthermore, the reaction mixture was stable for 30 min. Consequently, incubating the drug with the C-dots for a prolonged time was found to have no impact on the C-dots quenching, thus made the reaction simpler and faster.

### Validation

The International Council on Harmonisation (ICH) Q2(R)1 guidelines on validation of analytical procedures were followed to validate performance of the proposed method^53^. The validation parameters are displayed in Tables 1 and S1 in supplementary information.

**Table 1:** Validation parameters for the determination of NTX using the developed spectrofluorimetric method.

Analytical parameter	Proposed spectrofluorimetric method values
λ emission	416 nm
λ excitation	345 nm
Linearity range (µg/mL)	15 × 10^–3^–15.00
Intercept (a)	46.39
Slope (b)	45.03
Standard deviation of the intercept (S_a_)	3.07
Standard deviation of the slope (S_b_)	0.46
RSD% of the slope (S_b_ %)	1.11
Correlation coefficient (r)	0.9992
Standard deviation of residuals (S_y/x_)	6.74
Variance ratio (F)	9285.00
Significance F	3.42 × 10^–12^
LOD (μg/mL)	56.00 × 10^–4^
LOQ (μM)	15.00 × 10^–3^

#### Linearity and concentration range

In order to assess the linearity of the proposed methods, varying concentrations of the drug were analyzed using the optimum conditions mentioned above. The C-dots fluorescence intensity was measured over the concentration range; 15 × 10^–3^–15.00 µg/mL. Figure 4 demonstrates the resulting quenching of C-dots fluorescence upon addition of various concentrations of NTX. The least-squares method was applied for regression analysis and different values like correlation coefficients (r), intercepts (a), slopes (b), standard deviation of the intercept (S_a_) and slope (S_b_) were calculated. Table 1 presents all the statistical values for the proposed method. Results show that good linear calibration graph was generated; this was verified by the large values of the correlation coefficients (correlation coefficient value > 0.999) and the RSD% of the slope (S_b_%) that did not exceed 2%. Furthermore, the low significant F value confirms the low scatter of experimental points around the line of regression. Similarly, the small residual standard deviation (S_y/x_) proves that the plotted points are very close to the straight line which confirms the good linearity of the proposed method.

#### Limits of detection and quantification

Limit of detection (LOD) and limit of quantification (LOQ) were computed using the equations provided by the ICH guidelines. Where LOD = 3.3 S/b and LOQ = 10 S/b, where S is the standard deviation (SD) of six blank solutions (i.e. six C-dots solutions) and b is the slope of the calibration curve. The low calculated values for the LOD and LOQ indicate good sensitivity of the proposed method (Table 1).

#### Accuracy and precision

Three separate concentrations were analyzed using three replicate determinations for each within the same day to examine intra-day precision and accuracy. Similarly, the inter-day precision and accuracy were examined by analyzing the same three concentration levels using three replicate determinations for each, repeated on three consecutive days. Accuracy of the method was confirmed by the satisfactory percentage recovery (% Recovery) (99–101.4%) and the small values of percentage relative error (%E_r_) which did not exceed 2%. Also, the precision of the methodology was assessed and proven by the low values of percentage relative standard deviation (%RSD) which did not exceed 2.0% emphasizing the high reproducibility and accuracy of the developed method for estimation of NTX in bulk form (Table S1).

#### Selectivity and specificity

The selectivity of the proposed method was studied by observing the quenching effect of some co-administered drugs, as, ribavirin and remdesivir, which are used for the treatment of COVID-19^9–11^ and clarithromycin which is used in management of H-pylori infection^54^. Upon applying the proposed method, all the tested compounds showed no quenching effect to the used C-dots. Moreover, the normally found excipients and additives found in the pharmaceutical preparations did not interfere in the proposed methods as shown by the good percentage recoveries illustrated in Table 2. These studies support the selectivity and specificity of the developed methodology.

**Table 2:** Application of the developed spectrofluorimetric method for determination of NTX in Nanazoxid® oral suspension and tablets. (n = 5).

Nanazoxid® film coated tablets (500 mg)			Nanazoxid® oral suspension (100 mg/ 5 mL)		
Mean % recovery ± SD	RSD %	E_r_%	Mean % recovery ± SD	RSD %	E_r_%
100.13 ± 1.83	1.83	0.13	98.90 ± 1.35	1.37	− 1.10

### Application to pharmaceutical formulation

The proposed procedure was applied for the assay of NTX in the dosage forms available in the local market. Nanazoxid^®^ film coated tablets (500 mg) and Nanazoxid^®^ oral suspension (100 mg/ 5 mL) were estimated quantitatively using the proposed fluorimetric method. Percentage recoveries were calculated using external standard method. Good assay results showed acceptable accuracy and precision as illustrated by recovery%, SD, RSD % and E_r_% values presented in Table 2. The dosage forms, other coformulated excipients and additives had no impact on NTX assay. Consequently, in the presence of other co-formulated ingredients, the developed method demonstrated adequate specificity and reliability.

### Analysis of NTX and its metabolite (TX) in spiked human plasma

**Figure 5:**
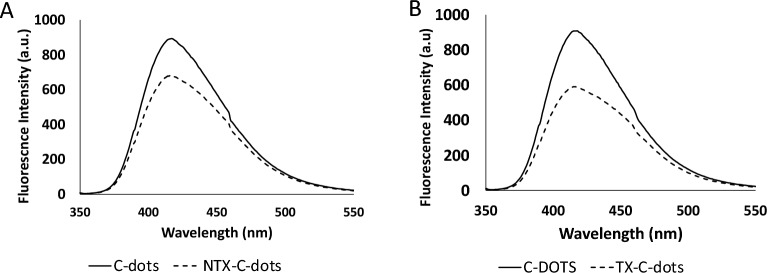
(**A**) Fluorescence emission spectra of 0.1 mL C-dots in aqueous solution upon addition of 2 μg/mL NTX spiked in human plasma and (**B**) Fluorescence emission spectra of 0.1 mL C-dots in aqueous solution upon addition of 2 μg/mL TX spiked in human plasma.

The method was successfully applied for the analysis of both NTX and TX in spiked human plasma. Figure 5 demonstrates the resulting quenching of C-dots fluorescence upon addition of human plasma samples spiked with 2 μg/mL NTX (Fig. 5a) and 2 μg/mL TX (Fig. 5b). Under the experimental conditions described above, a linear correlation was demonstrated by plotting the difference in fluorescence intensity versus the concentration of each compound. Upon subjecting the data to linear regression analysis, the following equations were obtained:

$${\text{FI}} = { 736}.{75} + { 5}.{48}0{\text{7 C}}\quad \quad {\text{r }} = \, \left( {0.{999}} \right)\;{\text{for}}\;{\text{NTX}}$$

$${\text{FI}} = {655}.{41} + {15}.{\text{452 C}}\quad \quad {\text{r }} = \, \left( {0.{998}} \right)\;{\text{for}}\;{\text{TX}}$$

where: FI is the difference in fluorescence intensity between the blank (i.e. C-dots) and the C-dots with either NTX or TX added, C is the drug concentration (μg/mL) and r is the correlation coefficient. High correlation coefficients values indicate good linearity of the calibration curves. Statistical analysis of the data demonstrated low relative error% (Er%) as shown in Table 3.

**Table 3:** Assay results for spectrofluorimetric determination of NTX and TX in spiked human plasma. (n = 9).

Concentration (µg/mL)	TX				NTX			
	%Recovery	Mean ± SD	RSD	Er%	%Recovery	Mean ± SD	RSD	Er%
0.025	102.18	100.24 ± 2.25	2.25	0.24	95.79	98.38 ± 2.11	2.14	− 1.61
0.025	101.45				98.38			
0.025	97.08				100.97			
0.2	99.45	99.14 ± 1.13	1.14	− 0.85	103.55	101.94 ± 1.32	1.29	1.94
0.2	100.36				100.32			
0.2	97.62				101.94			
1	100.36	98.54 ± 1.48	1.51	− 1.45	100.32	100.32 ± 2.64	2.63	0.32
1	98.54				103.55			
1	96.71				97.08			
3	103.4	102.39 ± 1.88	1.83	2.39	101.4	101.40 ± 1.76	1.73	1.4
3	99.75				99.24			
3	104.01				103.55			

The performance of the proposed method was demonstrated by its applicability to human plasma spiked with four different concentrations of each compound and determined using the corresponding regression equation. The assay results for NTX and TX determination in spiked plasma using the proposed fluorimetric protocol are summarized in Table 3.

### Assessment of method greenness

Nowadays, there is considerable interest in the impact of chemical processes on the environment and health. To completely eliminate any potential environmental risks, it was vital to establish the analytical methodologies' greenness. We employed the Eco-Scale estimating tool, which is a semi-quantitative technique that depends on the determination of the penalty points agreed upon with the Globally Harmonized System (GHS) for chemical labelling, occupational hazard, treatment method, energy consumption and waste volume. The total penalty points are deducted from "100", which represents the best greenness value. The proposed method calculated score was 87, revealing a perfect greenness value (Table 4)^47^. Recently, a different method (AGREE assessment) has been utilized to provide an impartial assessment of the environmental impact of the proposed methodology^48^. To represent the 12 principles of green analytical chemistry, AGREE provides a clock-shaped graph with 12 separated portions. Separately, each part refers to one principle that colored red, yellow, green according to the extent of greenness of the analytical procedure. Total assessment value is displayed in the center of the AGREE graph on a scale from 0 to 1. (Table 4). Both AGREE and Eco-greenness Scale's assessments supported the same findings.

**Table 4:** Collective comparison of the developed method and other reported spectrofluorimetric methods for NTX analysis.

Method	Condition	Linearity range	Quantum yield	LOD	Greenness assessment	
The proposed method	Quenching of the fluorescence of C-dots prepared from citric acid and l-cysteine using deionized water as diluting solvent at 345/416 nm	0.015–7 µg/mL	72%	0.005 µg/mL	Analytical Eco-scale	
					Reagent	Penalty points
					water	0
					L-cysteine	1
					Citric acid	1
					Sodium hydroxide	2
					Acetonitrile	4
					Instrument	Penalty points
					Spectrofluorimeter (< 0.1 kWh per sample)	0
					Hydrothermal autoclave reactor (1.5 kWh per sample )	2
					Occupational hazard (analytical process hermitization)	0
					Waste	3
					Total penalty point	13
					Total score	87
					AGREE metric approach	
Abdel-Lateef et al.^20^	Oxidizing NTX into a highly fluorescent product by sodium hypochlorite	1.0–5.0 µg/mL	–	0.143 µg/mL	Analytical Eco-scale	
					Reagents	Penalty points
					Ferric chloride	2
					Ferrous sulphate	1
					Cobalt chloride	6
					Ammonia	8
					Sodium hypochlorite	4
					DMF	12
					Instrument	Penalty points
					Spectrofluorimeter (< 0.1 kWh per sample)	0
					Heating water bath (1.5 kWh per sample )	2
					Occupational hazard (analytical process hermitization	0
					Waste	3
					Total penalty points	38
					Analytical Eco-Scale total score	62
					AGREE metric approach	
Quandeel et.al.^21^	Quenching of the fluorescence of C-dots prepared from cabbage and onion in BRB (pH = 8) at 340/418 nm	0.077–15.36 µg/mL	15.2%	0.022 µg/mL	Analytical Eco-scale	
					Reagents	Penalty points
					Boric acid	2
					Acetic acid	4
					Orthophosphoric acid	4
					Sodium hydroxide	2
					Instrument	Penalty points
					Spectrofluorimeter (< 0.1 kWh per sample)	0
					Microwave (< 1.5 Kwh per sample)	1
					Occupational hazard (analytical process hermitization	0
					Waste	3
					Total penalty point	16
					Analytical Eco-Scale total score	84
					AGREE metric approach	

### Comparison with reported spectrofluorometric methods

Abdel-Lateef et al. reported a spectrofluorometric method depending on oxidizing NTX (non-fluorescence) into a highly fluorescent product by sodium hypochlorite for the quantification of NTX^20^. While, Qandeel et al.^21^ have used plant synthesized quantum dots for the analysis of NTX in capsules dosage forms. Table 4 demonstrates a comparison of our proposed method to other previously published methods. Results show that our suggested method is highly sensitive. Such high sensitivity permitted the selective determination of TX, the main metabolite of NTX, in human plasma samples making this study the first spectrofluorimetric method in literature that determine TX in human plasma samples. In addition, the proposed procedure offers more advantages of being inexpensive, simple and no tedious multi-step procedures are required. Moreover, the suggested method does not require expensive instrumentation or complex analytical reagents. Additionally, applying water as the diluting solvent allows the developed method to be a greener substitute of the other previously reported methods^20,21^ as shown in Table 4. Thus, proving that our proposed method is more ecofriendly. Also, the proposed method can be beneficial in testing drug purity and routine quality control analysis. Moreover, it offers maximum sensitivity without the need for difficult or expensive instrumentation.

## Conclusion

The combination between using spectrofluorimetric technique and quantum dots nanosensor gives a highly sensitive, rapid and feasible method for the sensitive determination of NTX within concentration range of 15 × 10^–3^–15.00 µg/mL. It was possible to produce nitrogen and sulfur-doped CQDs with safe and high yields that had a lot of nitrogen and sulphur on their surface by hydrothermally reacting an aqueous solution of citric acid with l-cysteine. C-dots act as desirable luminescent nanosensors for the selective and sensitive determination of NTX. Depending on the need for very small amounts of organic solvents and the usage of water as a diluting solvent, the developed method is highly ecofriendly and green. The native C-dots fluorescence was efficiently quenched as a result of complementary overlaps of the NTX absorption band with the excitation fluorescence spectra of C-dots, resulting in an inner filter effect. inner filter effect was proved to be the underlying molecular mechanism of quenching. The obvious advantages of the validated proposed method are greenness, simplicity, reliability and low cost. Furthermore, the method demonstrated high sensitivity which allowed the determination of NTX in various pharmaceutical dosage forms without any pre-treatment or influence from the common excipients. Besides, this method is considered to be the first spectrofluorimetric method that determines both NTX and its main metabolite TX, in human plasma samples with high selectivity. Therefore, the proposed luminescent nanosensors can be considered superior to the previously reported spectrofluorimetric methods.

### Supplementary Information

Supplementary Information.

## Data Availability

All data will be available upon request. The corresponding author should be contacted for any data required for the conducted study.
