# Supplementary material for: Green “turn-off” luminescent nanosensors for the sensitive determination of desperately fluorescent antibacterial antiviral agent and its metabolite in various matrices
Source: Sci Rep. 2023 Aug 29;13:14131. doi: 10.1038/s41598-023-40946-4 (PMC10465507; doi:10.1038/s41598-023-40946-4)
Supplement: Supplementary file 1 — Supplementary Information. [file 41598_2023_40946_MOESM1_ESM.docx]

**Green “Turn-Off” luminescent nanosensors for the sensitive determination of desperately fluorescent antibacterial antiviral agent and its metabolite in various matrixes**

**Electronic Supplementary Information**

**Hadil M. Elbardisy^a^, Mai M. Elnaggar^b^, Tarek S. Belal^b^, Mahmoud A. Ragab^c^ and Amira F. El-Yazbi^b*^**

^a^Pharmaceutical Analysis Department, Faculty of Pharmacy, Damanhour University, Damanhour, 22511, Egypt

^b^Department of Pharmaceutical Analytical Chemistry, Faculty of Pharmacy, Alexandria University, Alexandria 21521, Egypt

^c^Department of Pharmaceutical Chemistry, Faculty of Pharmacy, Damanhour University, Damanhour, Buhaira 22516, Egypt

^*^Corresponding Author, Tel.: +20 34871317; fax: +20 34873273. E-mail address: [elyazbiamira@gmail.com](mailto:elyazbiamira@gmail.com)

**Figure S1.** Effect of **(a)** different pH of 2 mL 25 mM borate buffer**, (b)** different pH of 2 mL 25 mM phosphate buffer and **(c)** different volumes of 25 mM borate buffer, pH 7 on the quenching efficiency of NTX to C-dots.

**Figure S2.** Effect of different concentrations of **(a)** cetrimide (0.002 – 0.008 M), **(b)** SLS (0.005 – 0.02 M) and **(c)** tween (0.2 – 0.8%) on the quenching efficiency of NTX to C-dots.

**Figure S3.** Effect of **(a)** various diluting solvents and **(b)** reaction time on the quenching efficiency of NTX to C-dots.

**Table S1.** **Precision and accuracy for the spectrofluorimetric method proposed for the determination of NTX.** **(n = 9)**

| **Precision & Accuracy** | **Nominal**  **(µg/mL)** | **Found (µg/mL) ± SD** | **%RSD** | **%E_r_** |
| --- | --- | --- | --- | --- |
| **Within-day** | 1.00 | 1.01 ± 0.02 | 1.98 | 1.00 |
|  | 0.40 | 0.40 ± 0.005 | 1.25 | 0.00 |
|  | 5.00 | 5.07 ± 0.04 | 0.78 | 1.38 |
| **Between-day** | 1.00 | 0.99 ± 0.01 | 1.01 | -1.01 |
|  | 0.40 | 0.40 ± 0.007 | 1.75 | 0.00 |
|  | 5.00 | 5.01 ± 0.09 | 1.79 | 0.19 |
